# Management of a Flail Chest Caused by Multiple Costosternal Fractures: A Case Report

**DOI:** 10.7759/cureus.51082

**Published:** 2023-12-25

**Authors:** Zachary Taylor, Jeremy Miller, Danielle Z Azani, Brian Patterson, Andrew McCague

**Affiliations:** 1 Medicine, College of Osteopathic Medicine of the Pacific - Northwest, Western University of Health Sciences, Lebanon, USA; 2 General Surgery, Desert Regional Medical Center, Palm Springs, USA; 3 Surgery, Desert Regional Medical Center, Palm Springs, USA; 4 Surgery, Arrowhead Regional Medical Center, Colton, USA; 5 Trauma and Acute Care Surgery, Desert Regional Medical Center, Palm Springs, USA

**Keywords:** thoracic trauma, flail chest, surgical rib fixation, rib fracture, costochondral separation

## Abstract

Costochondral separation is a rare consequence of blunt thoracic trauma and can lead to life-threatening complications such as a flail chest. The diagnosis of costochondral separation remains challenging due to the obscurity of the condition on chest radiographs. Surgical rib fixation is a viable treatment option and research regarding its effectiveness and long-term benefits is promising but still evolving. Here, we discuss a case of flail chest caused by multiple costosternal fractures definitively managed with surgical rib fixation.

## Introduction

A flail chest is a life-threatening condition characterized by multiple rib fractures that result in a segment of the rib cage becoming free-floating. This condition is often a result of blunt chest trauma from high-impact accidents, falls, or industrial accidents [1]. The energy transferred to the chest during these traumatic events can lead to multiple rib fractures, causing the detachment of the flail segment. The clinical consequences of a flail chest are serious, primarily impairing chest wall stability and the mechanics of respiration. 

Rib fractures at the costosternal junction can lead to a phenomenon known as costochondral separation, in which one or more ribs detach from the sternum, resulting in a flail chest. The research surrounding this injury pattern and its management is limited, most likely due to its rare occurrence. Surgical rib fixation has been shown to effectively treat multiple rib fractures and flail chest in patients with costochondral separation [2-5]. Complications and outcomes associated with the procedure are subjects of ongoing research. In this case report, we present a patient with a flail chest as a result of costochondral separation and detail the use of surgical rib fixation as a treatment option. 

## Case presentation

A 58-year-old male presented to our Level I Trauma Center complaining of bilateral chest wall pain and difficulty breathing after suffering a 20-foot fall. It is unclear where he was climbing, but the patient was found next to a dumpster that he may have fallen into or next to. 

**Figure 1:**
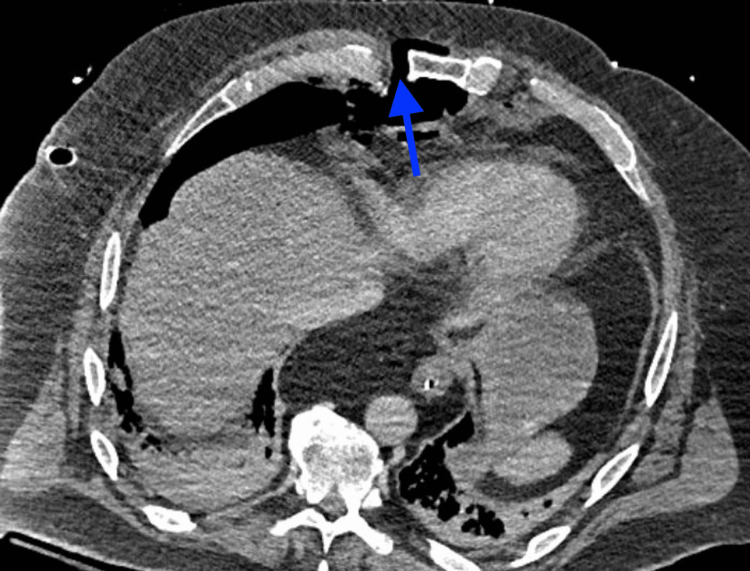
Axial chest CT on day one showing costochondral separation (blue arrow).

A primary and secondary survey was performed by the trauma team. On arrival, the patient was afebrile, the heart rate was 100 bpm, blood pressure was 121/85 mmHg, respiratory rate was 26, and oxygen saturation was 93% on room air, requiring 15 liters of supplemental oxygen. He was also intubated for respiratory failure and airway protection. Extended focused assessment with sonography in trauma (eFAST) exam was positive for decreased lung sliding on the right, so a chest tube was placed. He was also found to have a large 15 cm scalp laceration which was closed in the ED. He was taken to radiology for a CT for additional workup, with the finding that ribs two through seven on the right fractured and displaced over 1 cm at their costochondral and sternal chondral junctions (Figure 1). The CT also revealed a mediastinal hematoma, pneumomediastinum, a right pneumothorax, T1-3 spinous process fractures, and an L1 vertebral body fracture. After CT, he returned to the trauma bay and was hypotensive with systolic blood pressure in the 70s. The patient improved hemodynamically after the administration of two units of packed red blood cells and 1 liter of normal saline. He was then taken to the ICU for additional resuscitation. 

Admission complete blood count (CBC) and basic metabolic panel (BMP) are shown in Table 1 and Table 2, respectively. The patient’s alcohol level was negative and the urine drug screen was positive for methadone. 

**Table 1:** CBC on admission. CBC, Complete blood count

Blood Component	Laboratory Values	Reference Range
White blood cells	14,500/mm^3^	4,500-11,000/mm^3^
Hemoglobin	14.5 g/dL	Male: 13.5-17.5 g/dL
Hematocrit	43.2%	Male: 41%-53%
Platelets	246,000/mm^3^	150,000-400,000/mm^3^

**Table 2:** BMP on admission. BMP, Basic metabolic panel

Component	Laboratory Values	Reference Range
Sodium	134 mEq/L	135-145 mEq/L
Potassium	4.4 mEq/L	3.5-5 mEq/L
Chloride	104 mEq/L	95-105 mEq/L
Blood urea nitrogen	18 mg/dL	5-20 mg/dL
Creatinine	1.3 mg/dL	0.6-1.2 mg/dL
Carbon dioxide	20 mmHg	22-26 mmHg
Glucose	144 mg/dL	70-100 mg/dL

**Figure 2:**
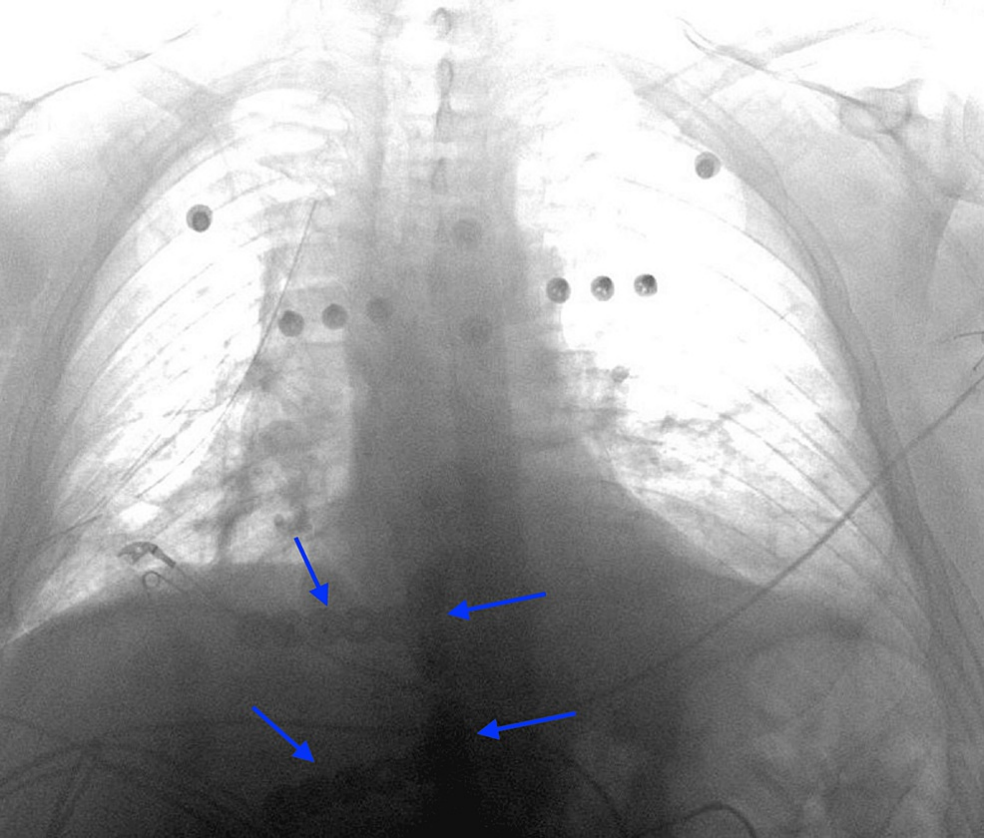
Postoperative chest radiograph showing titanium plates (blue arrows).

About 40 hours after arrival, once the patient was hemodynamically stable enough for surgery, he was taken to the operating room for chest wall stabilization. We decided to surgically reattach two of the seven dissociated ribs back to the sternum, anchoring the rib cage and allowing the other detached ribs to reattach nonoperatively. Using the DePuy Synthes MatrixRIB Fixation System (DePuy Synthes, Raynham, Massachusetts, United States), two T-shaped titanium plates were placed: one was screwed into the bone of the third rib and body of the sternum, while the other stabilized the fifth rib to the sternum in a similar manner (Figure 2). No other stabilizing devices were used. The chest wall was stable after fixation and a retained hemothorax was removed from the right thorax; two chest tubes were placed, then he was closed. 

He was extubated on postoperative day one and transferred to the medical-surgical unit the following day. The patient continued to improve and ambulated with physical and occupational therapy on the wards. His chest tubes were removed. He was discharged home on hospital day number twelve. He was seen in the clinic for a follow-up two weeks after surgery and had recovered well. 

## Discussion

Costochondral separation occurs following blunt thoracic trauma and is precipitated by multiple costochondral fractures resulting in the separation of costal cartilage from the sternum. This can lead to instability of the rib cage and potential compression of superior mediastinal structures [2]. Costochondral fractures are present in about 42% of patients who experience blunt chest trauma and are more common in elderly patients with calcification of the costal cartilage [6]. Symptoms associated with costochondral separation include severe chest pain, dyspnea, chronic cough, or paradoxical chest wall motion, depending on the site of injury. Patients may experience pain that worsens with rotational movements, making it particularly debilitating. The low radiopacity of costal cartilages and their inadequate depiction make costochondral separation inconspicuous on chest radiographs. Furthermore, sternal fractures, which are commonly associated with costochondral separation, can be challenging to identify as well due to their often transverse presentation. This leads to diagnostic challenges and underreporting in the medical literature [7]. Advanced imaging techniques such as MRI, CT, and ultrasonography are necessary to reliably diagnose and guide the management of costochondral separation and associated fractures [8,9]. 

Surgical rib fixation has emerged as a viable treatment option for patients with costochondral separation, especially in cases with severe symptoms or complications [2-4]. In such cases, surgical fixation can restore rib cage stability and alleviate the pain and distress associated with the condition, guiding patients’ return to normal physiologic function. The surgical procedure typically involves an anterior chest wall incision, debridement of damaged cartilage, and fixation of affected ribs to the sternum using specialized hardware [5,6]. 

Recent advancements in surgical techniques involve minimally invasive approaches to rib fixation, which may reduce surgical trauma and improve outcomes for patients with multiple rib fractures [5]. Video-assisted thoracoscopic surgery (VATS) has been used to provide a dynamic intraoperative evaluation of chest wall instability, plan the approach for fixation, and assess for associated injuries, which allows for direct minimally invasive repair. Although it is mainly diagnostic, adding VATS to surgical rib fixation for patients with costochondral separation is highly beneficial for therapeutic purposes as well, as it allows the evacuation of hemothorax, flushing of the thoracic cavity, and the administration of intercostal analgesia under direct vision [2]. 

It's essential to consider potential complications and outcomes associated with surgical rib fixation. While the overall risk of surgery- and implant-related complications is relatively low, it is crucial to monitor for complications such as wound and fracture-related infection. A systematic review of 48 studies focused on the incidence of rib fracture fixation complications and outcomes after the procedure. The study involved 1,952 patients and determined that the overall risk of surgical and implant-related complications was 10.3%, 2.2% of patients developed a wound infection, 1.3% of patients developed a fracture-related infection, and 30.9% of patients experienced pulmonary complications. Researchers also found that patients generally reported a good quality of life after the procedure [10]. 

Another study focused on short- and long-term outcomes of 166 patients who underwent surgical rib fixation. In the days to months after the procedure, 35% of patients developed pneumonia, 5% developed excess pleural fluid, and 3% developed implant-related infection. One year after the procedure, 48% of patients reported implant-related irritation and 9% had implant removal. Three years after the procedure, 62% of patients, on average, reported a good quality of life [11]. 

Research regarding the long-term outcomes of surgical rib fixation on patients' quality of life and implant-related complications is ongoing. But, in the case of costochondral separation, where symptoms can be disabling and severely affect a patient's daily life, rib fixation may offer substantial benefits in terms of pain relief and functional recovery. It's worth noting that, while surgical fixation does offer significant advantages in the treatment of costochondral separation, operative treatment is not always the first-line option [12]. The decision to pursue surgical intervention should be an informed one, considering the severity of symptoms, the patient's overall health, and the potential risks and benefits of the procedure. 

## Conclusions

Costochondral separation is a rare and underreported condition that presents diagnostic challenges due to its discreet nature on standard radiographs. Both classical and minimally invasive surgical rib fixation have emerged as effective treatment options for cases with severe symptoms or complications, providing pain relief and improved quality of life for patients. Research regarding complications and outcomes of these procedures is still ongoing. As more research and clinical experiences accumulate, the options for successful management of costochondral separation will continue to evolve, offering hope for improved outcomes and enhanced quality of life for affected individuals. 

## References

[1] Dogrul BN, Kiliccalan I, Asci ES, Peker SC (2020). Blunt trauma related chest wall and pulmonary injuries: an overview. Chin J Traumatol.

[2] Geraedts TC, Daemen JH, Vissers YL, de Loos ER (2021). Video-assisted thoracoscopic surgical rib fixation for costochondral separation injury. Innovations (Phila).

[3] George RJ, Stern HS (2014). An approach to surgical fixation of traumatic costosternal diastasis. ANZ J Surg.

[4] Prins JT, Wijffels MM (2021). Operative treatment of multiple costochondral dislocations in a patient with severe rib fractures and a flail chest following trauma. BMJ Case Rep.

[5] Bae CM, Son SA, Lee YJ, Lee SC (2023). Clinical outcomes of minimally invasive surgical stabilization of rib fractures using video-assisted thoracoscopic surgery. J Chest Surg.

[6] Ozen M, Cakmak V (2021). Prevelance of the costal cartilage fracture on the computerised tomography in chest trauma. Eur J Trauma Emerg Surg.

[7] Crandall J, Kent R, Patrie J, Fertile J, Martin P (2000). Rib fracture patterns and radiologic detection - a restraint-based comparison. Annu Proc Assoc Adv Automot Med.

[8] Subhas N, Kline MJ, Moskal MJ, White LM, Recht MP (2008). MRI evaluation of costal cartilage injuries. AJR Am J Roentgenol.

[9] Malghem J, Vande Berg B, Lecouvet F, Maldague B (2001). Costal cartilage fractures as revealed on CT and sonography. AJR Am J Roentgenol.

[10] Peek J, Beks RB, Hietbrink F (2020). Complications and outcome after rib fracture fixation: a systematic review. J Trauma Acute Care Surg.

[11] Beks RB, de Jong MB, Houwert RM (2019). Long-term follow-up after rib fixation for flail chest and multiple rib fractures. Eur J Trauma Emerg Surg.

[12] Hoepelman RJ, Beeres FJ, Beks RB (2023). Non-operative vs. operative treatment for multiple rib fractures after blunt thoracic trauma: a multicenter prospective cohort study. Eur J Trauma Emerg Surg.

